# Evaluation of Creatine Monohydrate Supplementation on the Gastrocnemius Muscle of Mice with Muscular Dystrophy: A Preliminary Study

**DOI:** 10.3390/pathophysiology32010002

**Published:** 2025-01-06

**Authors:** Victor Augusto Ramos Fernandes, Gabriela Pereira dos Santos, Amilton Iatecola, Daniela Vieira Buchaim, Ionaly Judith Faria Garcia, Carlos Henrique Bertoni Reis, Lívia Maluf Menegazzo Bueno, Bruna Trazzi Pagani, Rogerio Leone Buchaim, Marcelo Rodrigues da Cunha

**Affiliations:** 1 Postgraduate Program in Health Sciences, Faculty of Medicine of Jundiaí (FMJ), Jundiaí 13202-550, Brazil; victor.fernandes@ceunsp.edu.br; 2 Neurobiology Study Group, Nossa Senhora do Patrocínio University Center (CEUNSP), Itu 13300-200, Brazil; g290314@g.unicamp.br (G.P.d.S.); amilton.iatecola@ceunsp.edu.br (A.I.); 3 Graduate Program in Anatomy of Domestic and Wild Animals, Faculty of Veterinary Medicine and Animal Science, University of Sao Paulo (FMVZ/USP), Sao Paulo 05508-270, Brazil; danibuchaim@alumni.usp.br (D.V.B.); rogerio@fob.usp.br (R.L.B.); 4 Medical and Dentistry School, University Center of Adamantina (FAI), Adamantina 17800-000, Brazil; iojfgarcia@gmail.com (I.J.F.G.); likamaluf@usp.br (L.M.M.B.); 5 Beneficent Hospital (HBU), University of Marilia (UNIMAR), Marilia 17525-160, Brazil; dr.carloshenriquereis@usp.br; 6 Dentistry School, University of Marilia (UNIMAR), Marilia 17525-902, Brazil; brunatrazzi@usp.br; 7 Department of Biological Sciences, Bauru School of Dentistry (FOB/USP), University of Sao Paulo, Bauru 17012-901, Brazil

**Keywords:** Duchenne muscular dystrophy, creatine supplementation, creatine monohydrate, skeletal striated muscle, myopathy

## Abstract

**Background/Objectives:** Duchenne muscular dystrophy (DMD) is a genetic disease characterized by a lack of dystrophin caused by mutations in the DMD gene, and some minor cases are due to decreased levels of dystrophin, leading to muscle weakness and motor impairment. Creatine supplementation has demonstrated several benefits for the muscle, such as increased strength, enhanced tissue repair, and improved ATP resynthesis. This preliminary study aimed to investigate the effects of creatine on the gastrocnemius muscle in dystrophy muscle (MDX) and healthy C57BL/10 mice. **Methods:** Twenty MDX and C57Bl/10 mice were organized into groups and supplemented or not with creatine in a dosage of 0.3 mg for 8 weeks. Gastrocnemius tissue was analyzed using histomorphology and histomorphometric techniques. **Results:** The results demonstrated potential anti-inflammatory effects of creatine, with less observation of inflammatory infiltrates, the preservation of intramuscular glycogen, and reduction in tissue fibrosis in supplemented animals. **Conclusions:** These findings suggest that creatine may enhance tissue function and slow the progression of DMD. However, further research, with more analysis, is needed to elucidate molecular mechanisms underlying creatine’s effects on reducing mononuclear leukocytes and its role in mitigating tissue fibrosis.

## 1. Introduction

Duchenne muscular dystrophy (DMD) is a rare disease of genetic origin, with a higher incidence in men, with an incidence of 1 in every 5000 live births [1,2]. This progressive condition compromises the striated skeletal muscle tissue of the lower limbs, resulting in pseudohypertrophy. Pseudohypertrophy is characterized by an increase in myocyte volume without a corresponding increase in myofibrils, leading to cell weakening and motor impairment [3,4].

DMD is characterized by a severe reduction in the expression of dystrophin, an essential protein that anchors myofibrils and sarcolemma to the extracellular matrix. This marked absence of dystrophin leads to several muscle conditions, including dilated cardiomyopathy in cardiac tissue, pseudohypertrophy in lower limb muscles, and atrophy in the axial skeleton and upper limb muscles [5]. These conditions manifest in muscle tissue through several morphological changes, such as inflammation and local fibrosis. In addition, the metabolic context is severely impaired, mainly due to the reduction in intramuscular glycogen and the decreased availability of energy reserves [4,6,7].

Creatine monohydrate has the property of facilitating the phosphocreatine pathway, efficiently resynthesizing adenosine diphosphate (ADP) into adenosine triphosphate (ATP), especially in conditions of low oxygen availability, as well as in intense physical activity. The use of this supplement has been shown to be beneficial in several situations, such as hypoxia or the partial blockage of oxygen supply [8,9,10]. In addition, it can be described that the use of creatine monohydrate is greater and more reported than other isoforms or types of creatine mainly because this type is associated with water, penetrates muscle tissue more slowly, and enhances the chronic effects of its administration [11,12].

Creatine is synthesized endogenously in the liver, kidneys, and pancreas from the following amino acids: arginine, glycine, and methionine [13]. However, the exogenous supplementation of creatine monohydrate has gained prominence, mainly due to the difficulty in obtaining recommended amounts through endogenous production or diet alone. This supplementation has been shown to offer several benefits, including increased strength, improved tissue repair, and enhanced ATP resynthesis [14,15].

In addition to its positive effects on energy metabolism, creatine is also associated with a reduction in inflammatory infiltrates, as suggested by previous studies by the group [16]. Fernandes et al., in 2022, performed a study with nuclear morphometry, and a stereological analysis of the gastrocnemius and pectoralis major muscle tissues was conducted in MDX mice, supplemented or not with creatine for 16 weeks [15]. As a result, it was demonstrated that although the inflammatory progress caused by DMD was present in animals supplemented with creatine, this inflammation was less severe compared to that in the non-supplemented group. Additionally, another report examining the effects of creatine monohydrate on the diaphragm of MDX rats also revealing improvements related to the reduction in the inflammatory processes and tissue fibrosis [17]. These findings reinforce the hypothesis that creatine may be a beneficial supplement not only to improve muscular energy capacity, but also to attenuate inflammatory aspects associated with pathological muscular conditions [18].

Because creatine is a low-cost compound with varied benefits already documented in the literature, it is believed that its use in experimental models of muscular dystrophy could provide morphometric changes [19]. These changes may help to better understand the prescription of this amine in clinical conditions such as Duchenne muscular dystrophy. In view of the above, this study aimed to verify, through a preliminary preclinical protocol, with histomorphological and histomorphometric analysis, the effects of creatine supplementation on the gastrocnemius skeletal striated muscle between inbred mice of the X-linked muscular dystrophy lineage (MDX) and healthy C57BL/10 mice. Specifically, the research sought to answer the following questions:

- Does creatine supplementation influence the inflammatory infiltrate in dystrophic muscle tissue?
- What effects does creatine have on tissue fibrosis and muscle integrity in MDX mice?
- Can creatine supplementation interfere with energy metabolism by preserving intramuscular glycogen stores in dystrophic conditions?

We hypothesized that creatine monohydrate supplementation may reduce inflammation and fibrosis, along with improving the preservation of intramuscular glycogen in MDX mice. These results may further support the therapeutic potential of creatine not only to improve muscle energy metabolism but also to mitigate the pathological aspects of muscular dystrophy, such as chronic inflammation and tissue degeneration.

## 2. Materials and Methods

### 2.1. Experimental Draw and Ethical Aspects

This preliminary pre-clinical research was authorized by the Animal Use Ethics Committee of the Faculty of Medicine of Jundiaí (FMJ, Jundiaí, Brazil) under approval number 19/2021. A total of 20 male mice, aged 16 weeks, were utilized, comprising 10 MDX (dystrophic) mice and 10 C57BL/10 mice. The animals were divided into the following four groups:

- Group I included 5 C57BL/10 mice serving as controls for the study.
- Group II included 5 C57BL/10 mice that underwent supplementation with creatine monohydrate for a duration of eight weeks.
- Group III included 5 MDX mice supplemented with creatine monohydrate for a period of eight weeks.
- Group IV included 5 MDX mice that did not receive creatine monohydrate supplementation during the experiment.

All animals were four weeks old, and their body weights were correctly standardized at the beginning of the experimental protocol. The animals came from the Bioterium of the Biosciences Institute of the University of São Paulo (ICB/USP, São Paulo, Brazil) and were maintained during the experiment under standardized conditions in the Laboratory Animal Experimentation Sector (SEA-anatomy-registered at the Brazilian College of Animal Experimentation/COBEA and at the Brazilian Society of Laboratory Animal Sciences/SBCAL) of the Department of Morphology and Basic Pathology of the Faculty of Medicine of Jundiaí. This experimental study was performed in accordance with the ARRIVE (Animal Research: Reporting of In Vivo Experiments) guidelines.

The animals were provided with a solid diet and water *ad libitum* and housed in group cages (five mice of the same species per cage) under controlled conditions: a constant temperature of 23 °C and a 12 h light/12 h dark cycle, with lights on from 6:00 a.m. to 6:00 p.m. They were fed Labina^®^ (a standard chow diet for mice, provided by Purina, Paulínia, Brazil). The animals’ body weights (in grams) were recorded at both the start and end of the experimental period.

Euthanasia was performed one day after the final creatine supplementation, adhering to the standardized guidelines set by the ethics committee that approved the study. The procedure was conducted in a noise-free environment, away from other animals in the facility. Anesthetic agents, including Xylazine (Anasedan^®^, Ceva, Paulínia, Brazil), Ketamine (Dopalen^®^, Ceva, Paulínia, Brazil), and Thiopental (Thiopentax^®^, Cristália, Itapira, Brazil), were administered intraperitoneally in the lower left abdominal quadrant. Samples of the gastrocnemius muscle were collected in their entirety for analysis. No complications, such as illnesses or unexpected events, occurred that would have required the removal of any animal from the study.

### 2.2. Creatine Supplementation

Creatine monohydrate supplementation was provided exclusively to animals in Groups II and III. The supplement (Creatina Powder Creapure^®^, Creatine Monohydrate, Universal Nutrition, New Brunswick, NJ, USA) was administered at a dose of 0.3 mg per kilogram of body weight for a period of 8 weeks. The dosage was determined based on prior studies [20,21] that utilized similar supplementation in rodents, corresponding to doses used in humans to achieve ergogenic effects.

The supplementation was delivered via gavage, following the methodology outlined by Ramos Fernandes et al. (2022) [15]. An oro-esophageal probe (1 mm in diameter, 3 cm in length) adapted to a 3 mL syringe was used, with water serving as the infusion medium. Animals in groups II and III received supplementation on Mondays, Wednesdays, and Fridays, during the morning hours between 6:00 a.m. and 10:00 a.m. Meanwhile, animals in the other groups underwent the same gavage procedure but were only given water.

### 2.3. Histomorphological and Histomorphometric Analysis

After the experimental period and the euthanasia of the animals, the gastrocnemius muscle tissue was extracted and fixed in Bouin’s solution (a mixture of saturated aqueous picric acid—75 mL, formaldehyde—25 mL, and glacial acetic acid—5 mL) for 12 h to prepare it for processing and paraffin embedding. The tissue was subsequently rinsed in 70% alcohol and dehydrated through a graded alcohol series (80% alcohol twice, absolute alcohol three times, each for 1 to 2 h) before inclusion. The fragments were cleared in xylene for 1 to 2 h until they became translucent, then embedded in paraffin mixed with plastic polymers (Paraplast Plus^®^, Sigma-Aldrich, St. Louis, MO, USA) at 56 °C for approximately 1 h, followed by immersion in fresh paraffin at the same temperature. The tissues were arranged in plastic molds to obtain transverse histological sections.

The paraffin blocks were trimmed to create flat surfaces and sectioned into slices 5 μm thick. These sections were mounted on albumin-coated slides and placed in an oven at 60 °C. Once prepared, the slides were stained for various analyses: hematoxylin/eosin (HE) for general morphology, PAS (Periodic Acid–Schiff) for intramuscular glycogen detection, and Masson’s Trichrome for identifying collagen fibers. The prepared slides were then examined and photographed using a Nikon^®^ Eclipse E100 microscope (Tokyo, Japan) equipped with a Sony DSC-W120 imaging system (Sony^®^, Tokyo, Japan) at the Department of Morphology and Basic Pathology, Faculty of Medicine of Jundiaí, Brazil.

**Figure 1:**
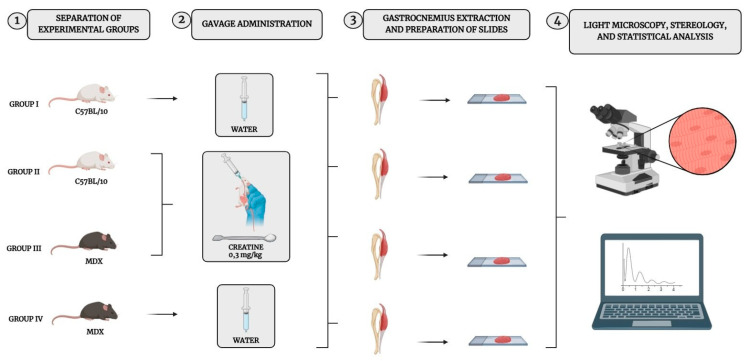
Schematic demonstration of the methodological process of the present study. The figure demonstrates the process of group separation, gavage administration, tissue extraction, slide preparation, microscopy, and statistical analysis.

Additionally, a portion of the tissue samples was deep-frozen, allowing the researcher to practice obtaining sections using a cryostat microtome at −20 °C. Measurements were conducted with the aid of a 10× eyepiece equipped with a micrometer scale and coupled to the Nikon^®^ Eclipse E100 microscope. Observations were documented using the 100× objective lens. The eyepiece was pre-calibrated with a specialized slide featuring 0.01 mm divisions, enabling the conversion of eyepiece units into micrometers. From these values, the average volumes of the nuclei were calculated using the following formula: V = 4/3 π r^3^ for spherical nuclei, with “r” being the radius of the nucleus and V = 4/3 π (d/2)^2^ D/2 for elliptical nuclei, with “d” being the smallest diameter and “D” being the largest nuclear diameter (Figure 1).

### 2.4. Statistical Analysis

We performed quantitative analysis by measuring the sarcoplasmic volume of myocytes in the gastrocnemius muscle, which expresses a general trend in relation to the variables of inflammatory infiltrate, areas of fibrosis, and local glycogen by stereological methods. This variable was compared between dystrophic mice (MDX), treated and not treated with creatine, by means of an unpaired Student’s *t*-test, *p* < 0.05, using the statistical program GraphPad Prism (GraphPad^®^ Software version 8.0, La Jolla, CA, USA).

## 3. Results

**Figure 2:**
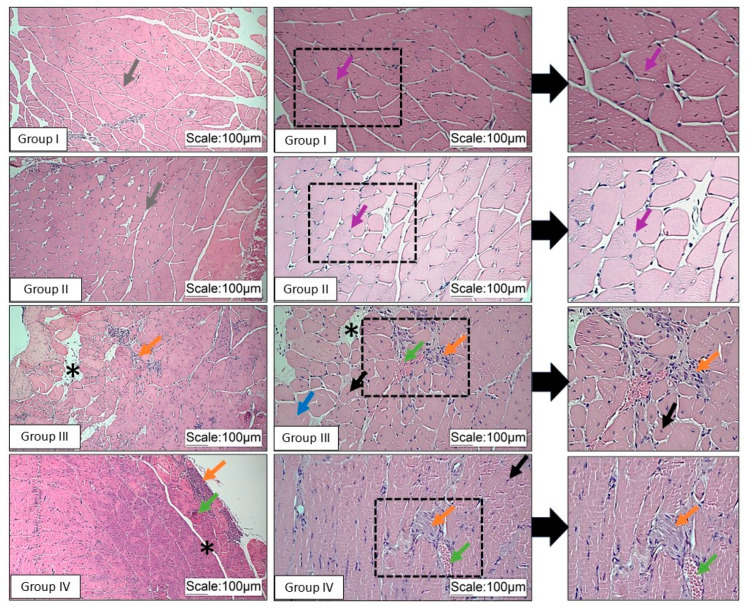
Histological images of the gastrocnemius muscle with hematoxylin eosin (HE) staining. Group I—healthy control group; Group II—healthy control group supplemented with creatine monohydrate; Group IV—MDX group; Group III—MDX group supplemented with creatine monohydrate. In GI and GII, the normal definition of the endomysium (gray arrow) can be observed, as well as organized muscle fibers with a peripheral nucleus (pink arrow). In GIII and GIV, there were tissue changes compatible with an inflammatory process due to the visualization of red blood cell congestion (green arrow) and presence of inflammatory infiltrate (orange arrow), centralization of the nucleus (black arrow), increased endomysial space (asterisk), suggesting fat infiltration (asterisk) and the presence of a pseudohypertrophic myocyte (blue arrow). In the image on the left, 10× objective; in the center, 20× objective, highlighting of the region marked in the grid on the right.

During the experiment, no unplanned events occurred that required reporting. Figure 2 demonstrates the comparison of histological sections of the gastrocnemius from the different experimental groups using a 10× and 20× magnification. The nucleus and sarcoplasm of the tissues were highlighted with hematoxylin and eosin staining.

Figure 2 presents a comparison between samples, allowing the identification of eccentric nuclei near the sarcolemma of myocytes in healthy groups, both treated and untreated with creatine (Groups II and I, respectively) and a compatible muscle volume between the samples of these groups. Frames C and D in the same figures demonstrate samples from dystrophic animals. Frame C displays MDX animals that were not treated, while Frame D shows MDX animals that were supplemented with creatine. Creatine was administered at a dosage of 0.03 g per kilogram of body weight for eight weeks, as outlined in the study’s experimental protocol. Both samples (Group III and Group IV) exhibited areas of inflammatory infiltration (indicated by white arrows), suggesting the presence of mononuclear leukocytes, especially in the perimysium.

**Scheme 1:**
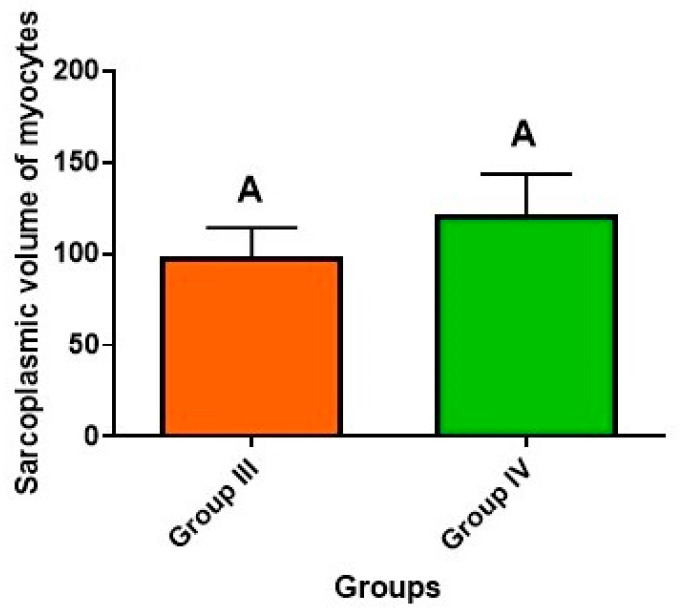
Quantitative analysis of the sarcoplasmic volume of myocytes in the gastrocnemius muscle. MDX group (Group IV) and MDX group supplemented with creatine monohydrate (Group III). Equal capital letters indicate no significant difference (A = A). *p* value = 0.0893.

The quantitative analysis presented in Table 1 and Scheme 1 indicates that the mean sarcoplasmic volume, which was affected by the inflammatory infiltrate, demonstrated lower values in the sample of animals supplemented with creatine monohydrate (MDX + Creatine, Group III), but without a statistically significant difference (*p* > 0.05). Histologically, in Figure 2, these aspects can be identified in the extent of the infiltration.

**Figure 3:**
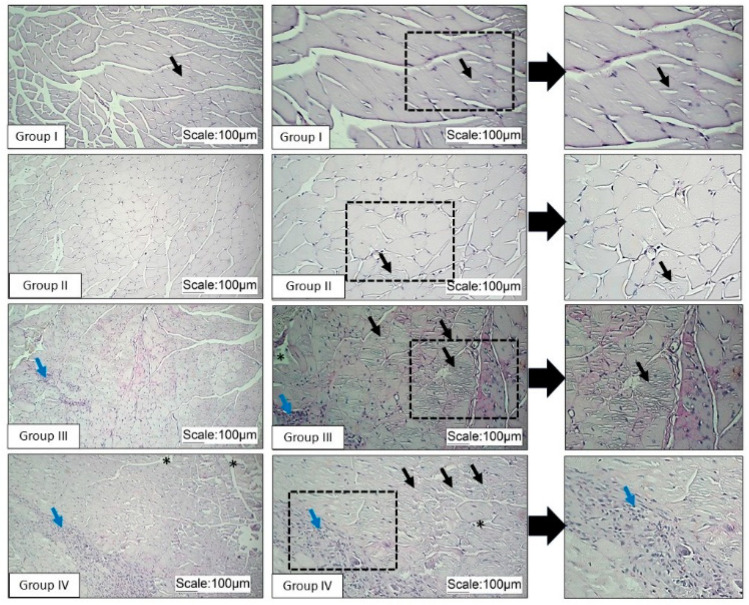
Histological images of the gastrocnemius muscle, stained by Periodic Acid–Schiff (PAS). Group I—healthy control group; Group II—healthy control group supplemented with creatine monohydrate; Group IV—MDX group; Group III—MDX group supplemented with creatine monohydrate. The presence of glucose polymers (black arrow) can be observed in the groups supplemented with creatine (II, III) more evidently when compared to the non-supplemented groups (I, IV). In the MDX groups, changes such as the presence of inflammatory cells (blue arrow) and increased endomysial space (asterisk) occurred. In the image on the left, 10× objective; in the center 20× objective, highlighting of the region marked in the grid on the right.

Figure 3 demonstrates the gastrocnemius of animals from the different experimental groups in this study, stained with Periodic Acid–Schiff (PAS), which is capable of associating with glycogen polymeric structures (glycogen) present in the sample’s sarcoplasm.

**Figure 4:**
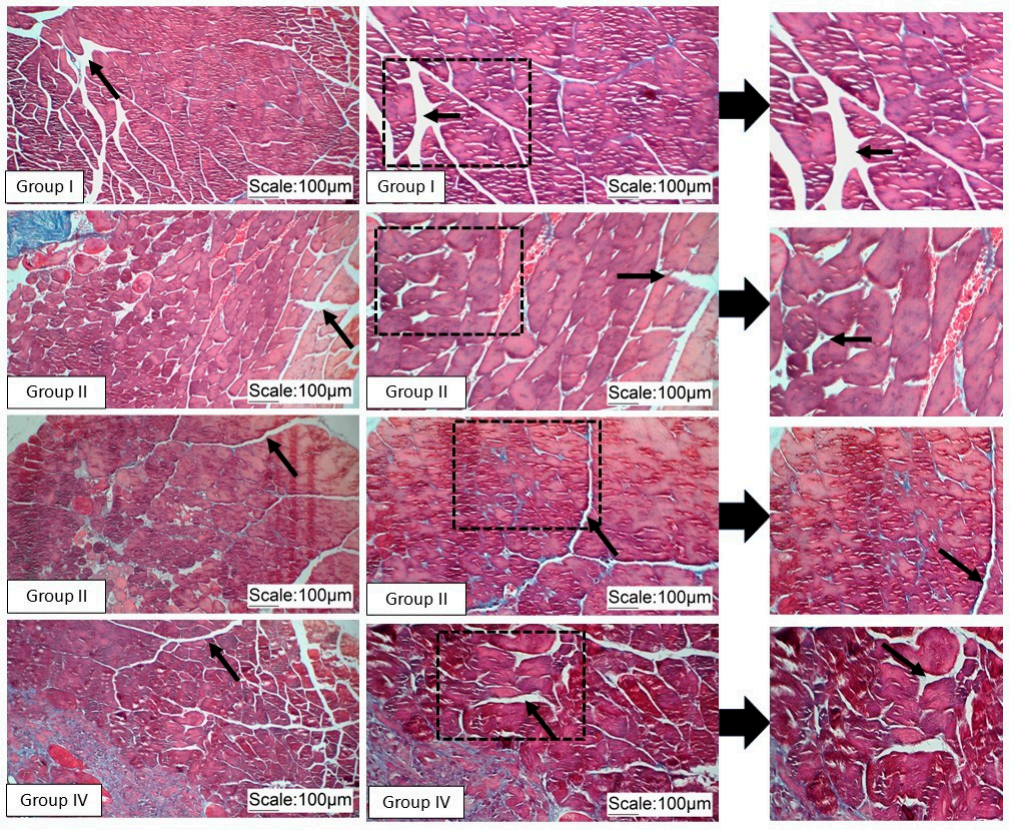
Histological images of the gastrocnemius muscle stained by Masson’s Trichrome. Group I—healthy control group; Group II—healthy control group supplemented with creatine monohydrate; Group IV—MDX group; Group III—MDX group supplemented with creatine monohydrate. Thin black arrows highlight connective tissue. In the image on the left, 10× objective; in the center, 20× objective, highlighting of the region marked in the grid on the right.

Figure 4 demonstrates the gastrocnemius of the animals stained with Masson’s Trichrome, which highlights the connective tissue and the relationship between this tissue and skeletal striated muscle.

Regarding the histomorphometry of sarcoplasmic volume, as previously demonstrated in Table 1 and Scheme 1, the comparative results between the diseased groups (MDX and MDX + creatine) in the study did not demonstrate a statistical difference between the samples evaluated, but the mean values were lower in the group with MDX animals treated with creatine. Figure 4 presents a comparison of the samples in a Masson Trichrome stain, in which the dye was attracted to collagen fibrils, therefore indicating areas of concentration of this protein (mainly indicated by the blue color). In this study, collagen primarily correlated with regions of tissue regeneration characterized by fibrosis. Figure 4 reveals that samples from dystrophic animals exhibited areas of tissue fibrosis, more prominently observed in the non-supplemented group. This observation aligns with the findings in Figure 2, which indicate the presence of inflammatory infiltrations in the tissue. Thus, when examining frames C and D of Figure 3, it becomes evident that samples displaying infiltration also exhibit regions of tissue fibrosis.

## 4. Discussion

This preliminary study aimed to analyze the effects of creatine supplementation on the gastrocnemius skeletal striated muscle between inbred mice of the X-linked muscular dystrophy lineage (MDX) and healthy C57BL/10 mice. Skeletal striated muscle tissue has a fusiform shape in its morphology with multiple nuclei positioned close to the sarcoplasmic membrane, in which the extracellular matrix remains anchored through proteins such as dystrophin, which establishes the proteins of the muscle cell’s cytoskeleton together with glycoproteins in the extracellular matrix, through the sarcoplasmic membrane [22,23,24].

**Figure 5:**
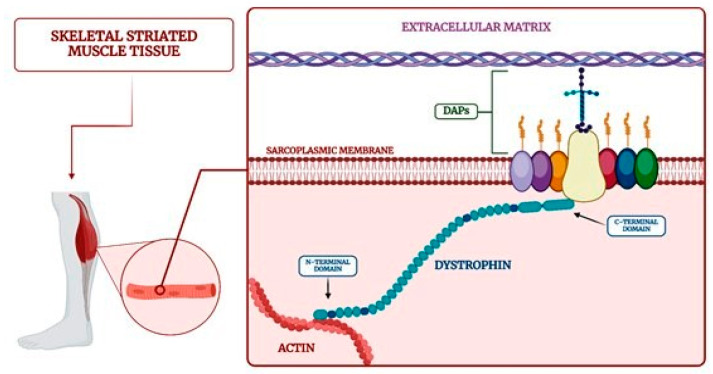
Schematic demonstration of protein interactions in skeletal striated muscle tissue. The figure demonstrates dystrophin interacting with the DAP complex and cytoskeletal proteins (actin).

Dystrophin’s main function is to act on muscle structure and function, comprising a protein with four domains, including an N-terminal linked to actin (myocyte cytoskeleton protein), a spectrin-like domain, a cysteine-rich domain, and a C-terminal, which remains attached to extracellular matrix glycoproteins, characterizing a complex known as dystrophin-associated proteins (DAPs) or dystrophin-associated glycoproteins (DAGs). In this complex, the presence of alpha-dystroglycan is observed, which has been identified as a receptor for agrin, a protein that induces the aggregation of acetylcholine receptors at the neuromuscular junction, resulting in muscle contraction [1,25]. Therefore, the complex established between dystrophin and other muscle cell stabilization molecules together with the extracellular matrix enables cell stability and assists in its functional process—contraction (Figure 5) [26].

In patients with Duchenne muscular dystrophy, a significant reduction in dystrophin is observed, an aspect associated with mutations in the DMD gene linked to the X chromosome. DMD is the largest known human gene, consisting of 79 exons and more than seven promoters [27]. Compared to other human genes, the mutation rate in the dystrophin gene is relatively high, with 1/3 of mutations occurring in non-inherited situations and 2/3 occurring in genetically inherited conditions [28]. The types of mutation can be gene deletions (65%), duplications (9%), small mutations (25%), and less common atypical mutations (<1%). In the present study, two contexts of gastrocnemius muscle physiology were compared: MDX mice with Duchenne muscular dystrophy and healthy C57/BL6 mice without any pathological processes, including in muscle tissue [29,30].

Nuclear centralization was another finding in our experimental protocol that was consistent with previously published literature on the subject, being an atypical characteristic in skeletal striated muscle tissue [21,31,32,33,34,35]. This condition was evidenced histologically, which showed samples subjected to PAS staining, revealing glycidic aggregations in the sarcoplasm of the cells. Regarding glycogen, PAS staining showed white dot markings in the sarcoplasm, indicating the presence of intramuscular glycogen. Therefore, the PAS staining applied in this study, among the groups, revealed a more visible presence of glycogen in the samples treated with creatine monohydrate for eight weeks, both in healthy animals (Group II) and in diseased animals (Group III) [36,37,38].

As previously explained, PAS staining applied in the analysis between groups revealed a higher presence of glycogen in the samples treated with creatine monohydrate for eight weeks, both in healthy animals (Group II) and in sick animals (Group IV). Glycogen increase indicates a greater energy reserve in the analyzed muscle tissue, supporting previous findings in the literature suggesting that creatine supplementation enhances glycogenesis in muscle tissue [39]. This effect is primarily attributed to the resynthesis of adenosine triphosphate via the phosphocreatine pathway and the optimization of glycolysis under metabolic conditions associated with both health and disease [40].

Duchenne muscular dystrophy affects muscle metabolism by reducing the stabilization of the sarcolemma, which compromises the stability of myofibrils and their association during muscle contraction [41]. This condition hinders muscle movement, requiring increased energy input and consumption through the aforementioned metabolic pathways (phosphocreatine and glycolysis) [42]. Therefore, creatine supplementation represents a promising strategy to preserve intramuscular glycogen, potentially enhancing energy conservation for tissue function [43,44].

Tissue fibrosis is recognized as a regenerative response characterized by the presence of mononuclear leukocytes, such as macrophages, which contain phagocytose cellular debris from regions affected by immune responses [45,46]. The formation of collagenous tissue as a substitute for compromised muscle tissue diminishes the quality of muscle contraction, resistance, and elasticity, leading to motor impairment and reduced tissue function [22]. Figure 4 indicates that although animals treated with creatine monohydrate for eight weeks exhibited tissue fibrosis, the extent of collagenous tissue was less pronounced compared to that of untreated animals. These findings suggest that creatine supplementation may contribute to greater long-term tissue preservation and improved motor function. Additionally, the morphometric analysis revealed significant differences in the sarcoplasmic volume of samples of DMD mice, with the volume being much higher in non-supplemented animals (121.6 µM) when compared to animals that received creatine supplementation (98 µM) for eight weeks.

Duchenne muscular dystrophy is characterized by a clinical sign known as pseudohypertrophy, predominantly affecting the gastrocnemius and soleus muscles in patients [47,48]. Pseudohypertrophy involves an increase in myocyte volume without the corresponding growth of myofibrils, leading to diminished functional capacity [49]. The observation of smaller gastrocnemius myocyte volumes in samples from MDX mice treated with creatine monohydrate suggests that the supplementation reduced the pseudohypertrophy of the cells. In addition to the other findings described, such as the less evident tissue fibrosis and qualitatively less evidence of inflammatory infiltrate in the perimysium, it is reasonable to suggest that creatine supplementation promotes beneficial effects on tissues by improving tissue function and delaying the aggressive progression of this genetic disorder.

As possible limitations of the study, we can consider that we focused exclusively on MDX mice, an animal model for Duchenne muscular dystrophy (DMD) that may not fully capture the complexity of the disease in humans, including the genetic and environmental variability that influences DMD progression. These findings have clinical and translational importance, as they indicate that creatine may improve tissue function and offer a promising therapeutic approach to mitigate the degenerative effects of DMD. Furthermore, the research highlights that creatine monohydrate, already widely used for ergogenic purposes, may have broader applications in clinical conditions such as DMD. The reduction of pseudohypertrophy and the preservation of muscle tissue observed in creatine-treated mice suggest additional benefits for muscle function and quality of life in patients with DMD.

As a future perspective, the qualitative results of this preliminary study on inflammation, glycogen, and fibrosis could be confirmed by more methods, such as RT-PCR or Western blotting.

## 5. Conclusions

This preliminary study investigated the effects of creatine monohydrate in dystrophic MDX mice over eight weeks, using a dosage of 0.03 g per kilogram of body weight. Despite the non-significant quantitative difference between the dystrophic animals, the not supplemented ones (Group IV) in relation to the supplemented ones (Group III), there was a lower mean volume of sarcoplasm in the supplemented ones, an important reference for evaluating inflammation, fibrosis, and the preservation of intramuscular glycogen. From this, new studies may confirm that, in addition to its known ergogenic benefits, creatine may offer therapeutic potential in the treatment of dystrophic muscle degeneration, contributing to tissue integrity and modulating inflammatory processes.

## Figures and Tables

**Table 1:** Quantitative analysis of the sarcoplasmic volume of myocytes in the gastrocnemius muscle.

Group	Mean	Standard Deviation	*p* Value
MDX (IV)	121.6	21.9	0.0893
MDX + creatine (III)	98.0	16.3	0.0893

Comparison between the samples after applying an unpaired Student’s *t*-test, mean and standard deviation, *p* < 0.05. There was no significant difference between the groups analyzed.

## Data Availability

All quantitative data of the results will be made available by the authors upon request.
